# Lens hardness not related to the age-related decline of accommodative amplitude

**Published:** 2007-06-27

**Authors:** Ronald A. Schachar, Barbara K. Pierscionek

**Affiliations:** 1 Department of Physics, University of Texas at Arlington, Arlington, TX; 2 Department of Biomedical Sciences, University of Ulster in Coleraine, Coleraine, Northern Ireland

Using atomic force microscopy, Ziebarth et al. [1] measured the Young's modulus of the center of intact *fresh* monkey lenses from donors 4.2 to 10 years of age. Young's modulus is a material property of the lens that is directly related to its hardness. From the data given in Table 2 of the author's paper, we calculated the coefficient of determination and found Young's modulus of the lens, and therefore lens hardness, does not appear to be related to age, R^2^<0.03. During this period of life, monkey accommodative amplitude declines linearly by approximately 7 diopters [2]. Therefore, the authors' data, in contrast to studies of non-fresh lenses [3], demonstrates that lens hardness is not related to the age-related decline in accommodative amplitude.

Consistent with this conclusion, in vitro experiments that evaluated *fresh* human postmortem crystalline lenses from donors less than 40 years of age similarly found that there was no change in lens hardness with age [4]. Moreover, in vivo measurements of optical density [5] and lens fluorescence [6], which are associated with lens hardness, have not been found to correlate with accommodative amplitude, and the speed of ultrasound through the lens in vivo does not change with age [7].

While experiments that have evaluated the viscoelastic properties of thawed lenses following freezing at -70 °C in liquid nitrogen have observed an age-related increase in nuclear stiffness [8-11], these observations are subject to concern over the collection and preservation of the tissue. It has been shown that:

1. Cold cataracts form predominately in the nucleus [12];
2. The severity of cold cataract is age dependent [13];
3. Freezing improves the transparency of cortical cataracts but does not affect nuclear cataracts [14]; and
4. Freezing alters the distribution of free and bound water within the lens [15].

Since freezing can affect the protein structure of the human lens and will, consequently, affect the shear moduli of the lens cortex and nucleus differently, depending on the age of the donor tissue, it is unlikely that these studies accurately reflect in vivo lens stiffness. Interestingly, even these altered frozen lenses, from donors less than 25 years of age, show no demonstrable changes in lens stiffness with age [16].

The authors' study of *fresh* primate lenses further confirms that lens hardness is not responsible for the age-related decline in accommodation that eventually results in the clinical manifestation of presbyopia [17]. The etiology of the age-related decline in accommodation is normal equatorial lens growth [18].
